# High-luminance perovskite light-emitting diodes with high-polarity alcohol solvent treating PEDOT:PSS as hole transport layer

**DOI:** 10.1186/s11671-018-2505-6

**Published:** 2018-04-27

**Authors:** Mengge Wu, Dan Zhao, Zijun Wang, Junsheng Yu

**Affiliations:** State Key Laboratory of Electronic Thin Films and Integrated Devices, School of Optoelectronic Science and Engineering, University of Electronic Science and Technology of China (UESTC), Chengdu, 610054 China

**Keywords:** Perovskite light-emitting diodes, PEDOT:PSS, Alcohol solvent treatment, Polarity, Luminance

## Abstract

Perovskite light-emitting diodes (PeLEDs) are fabricated with a structure of indium tin oxide (ITO)/poly(3,4-ethylenedioxythiophene):polystyrene sulfonate (PEDOT:PSS)/CH_3_NH_3_PbBr_3_ (MAPbBr_3_)/1,3,5-tris(2-*N*-phenylbenzimidazolyl) benzene (TPBi)/Ag. PEDOT:PSS films treated with alcohols including methanol, ethanol, and isopropanol are used to realize high-performance PeLEDs. Among them, by using PEDOT:PSS film treated by methanol as the hole transport layer, the PeLED with a maximum luminance of 2075 cd m^−2^ and a maximum current efficiency of 0.38 cd A^−1^ is achieved. Meanwhile, the result shows that the luminance of PeLEDs increases with the polarity of alcohol solvent. The conductivity of PEDOT:PSS films and crystallization of perovskite films are analyzed to gain a deep illumination into the influence of alcohol solvent treatment on device performance. It is also found that the treatment brings not only an improved hole injection capability but also a significantly enhanced crystallization of perovskite. This work indicates that our founding brings a simple and effective method to improve the device performance of PeLEDs.

**Electronic supplementary material:**

The online version of this article (10.1186/s11671-018-2505-6) contains supplementary material, which is available to authorized users.

## Background

Organic-inorganic hybrid perovskite materials have attracted enormous research interest because of their excellent properties. These properties include low material cost, compatible with solution processing, superior carrier mobility, and tunable optical bandgap [1–5]. At the same time, perovskite materials have a narrow full width at half maximum (FWHM) and a high photoluminescence quantum yield (PLQY) [6–9]. These characters make perovskite materials become the promising candidates for information display and solid-state lighting source compared to organic light-emitting diodes [10, 11] and provide the premise for low-cost and roll-to-roll fabrication. In 2014, Friend and co-workers firstly reported a new perovskite light-emitting diode (PeLED) based on solution-processing organometal halide perovskite with a sandwich structure. In green PeLEDs, a maximum luminance of 364 cd m^−2^ and a maximum external quantum efficiency (EQE) of 0.1% were obtained [12]. Since then, many significant works have been carried out to study PeLEDs. In 2015, Tae-Woo Lee and co-workers boosted the current efficiency (CE) of PeLEDs to 42.9 cd A^−1^ by increasing the proportion of methylammonium bromide in perovskite precursor solution and using nanocrystalline pinning-process method in the process of spin-coating perovskite [13]. In 2016, Jianpu Wang and co-workers reported a PeLEDs based on self-organized multiple quantum wells, and they achieved a very high EQE up to 11.7% [14]. In 2017, Chih-Jen Shih and co-workers fabricated PeLEDs with a high PLQY up to 92% by adding low-dielectric-constant compound, poly(methyl methacrylate) (PMMA), into perovskite colloidal solution [15]. These previous works indicate that PeLEDs have a great development potential in high-performance aspect.

As well known, the frequently used device structure of PeLEDs is anode (on transparent substrate, i.e., light-output direction)/hole transport layer (HTL)/perovskite emission layer (EML)/electron transport layer (ETL)/cathode [16–19]. In this structure, poly(3,4-ethylenedioxythiophene):polystyrene sulfonate (PEDOT:PSS) is the most common hole transport material because of its high transparency in the visible range (380–760 nm) and compatible with solution processing [20, 21]. However, the hole injection capability from PEDOT:PSS layer to EML is low. The main reason for this is that there is a high hole injection barrier from pristine PEDOT:PSS layer to EML, which caused by the highest occupied molecular orbital (HOMO) of PEDOT:PSS layer (5.2 eV) is much shallower than the HOMO of perovskite layer (5.6–5.9 eV) [20–22]. This high hole injection barrier (0.4–0.7 eV) hinders hole injection into EML efficiently, thus leading to an imbalance of charge carriers in EML.

To alleviate this problem, lots of efforts have been made to reduce the hole injection barrier from PEDOT:PSS layer to EML. For example, Tae-Woo Lee and co-workers combined PEDOT:PSS with perfluorinated ionomer (PFI) as a self-organized buffer HTL [13, 23]. The HOMO of the buffer HTL (absolute value) increased gradually from the bottom surface (5.2 eV) to the top surface (5.95 eV). This gradual increase of HOMO level can facilitate hole injection into CH_3_NH_3_PbBr_3_ (MAPbBr_3_) more efficient than pristine PEDOT:PSS film. In green PeLEDs with a buffer HTL, a maximum luminance of 417 cd m^−2^ was achieved. Da Bin Kim and co-workers mixed PEDOT:PSS with MoO_3_ (PEDOT:MoO_3_) as a composite HTL to reduce the hole injection barrier [24]. When the amount of MoO_3_ powder in PEDOT:PSS dispersion solution is 0.7 wt%, the HOMO of PEDOT:MoO_3_ composite layer increased from 5.15 to 5.31 eV. But the addition of excessive MoO_3_ powder into PEDOT:PSS solution would decrease the efficiency of device, which is probably due to the non-uniform morphology of MAPbBr_3_ film caused by excessive MoO_3_. Although these methods can reduce hole injection barrier, they are all doping with new materials in PEDOT:PSS solution, which is not conductive to large-scale industrial fabrication. Therefore, there is an urgent requirement to develop a more convenient method.

In this work, a high-luminance PeLEDs with MAPbBr_3_ as the EML were fabricated by spin coating alcohol solvent on PEDOT:PSS films before annealing treatment. By analyzing the characteristics of methanol (MeOH), ethanol (EtOH), and isopropanol (IPA), it is found that the polarity of alcohol solvent is a dominant factor for the improvement of PeLEDs performance. Alcohols with high polarity can introduce a screening effect between positively charged PEDOT and negatively charged PSS, and so they can take away some insulator PSS from PEDOT:PSS during spin-coating process [20]. As a result, the hole injection capability from PEDOT:PSS to perovskite film is dramatically improved. Meanwhile, after treated by alcohols with high polarity, there is a smoother PEDOT:PSS film, which can help obtain smaller perovskite grains and better perovskite coverage by improving the surface energy of PEDOT:PSS film [25]. So MeOH with the highest polarity can greatly improve the maximum luminance of PeLEDs from 261 to 2075 cd m^−2^, and a maximum CE from 0.1 to 0.38 cd A^−1^.

## Methods

**Fig. 1:**
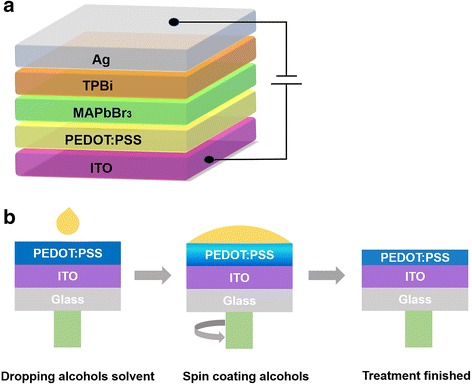
**a** Device structure of PeLEDs. **b** Process of spin coating alcohol solvent on PEDOT:PSS films

Properties of alcohol solvent used in this paper are presented in Table 1. The device structure of PeLEDs and experimental operation process are shown in Fig. 1. Device structure was indium tin oxide (ITO)/PEDOT:PSS/MAPbBr_3_ (70 nm)/1,3,5-tris(2-*N*-phenylbenzimidazolyl) benzene (TPBi) (40 nm)/Ag (100 nm). In this device structure, ITO and Ag were used as the anode and cathode, respectively, while PEDOT:PSS, MAPbBr_3_, and TPBi were used as the HTL, EML, and ETL, respectively. ITO substrates with a sheet resistance of 15 Ω/sq. were consecutively cleaned with water-detergent solution, acetone solvent, deionized water, and IPA solvent in ultrasonic bath each for 15 min. After dried in an oven, these cleaned ITO substrates were treated with oxygen plasma for 15 min. Then, PEDOT:PSS was spin-coated at 5000 rpm for 60 s on ITO substrate. For control samples, PEDOT:PSS/ITO substrates were annealed at 120 °C for 20 min directly without any treatment. For experiment samples, MeOH, EtOH, and IPA were spin-coated on PEDOT:PSS/ITO substrates at 5000 rpm for 30 s, respectively; then, these substrates were annealed at 120 °C for 20 min. After that, all of these substrates were transferred into a nitrogen glove box. The MAPbBr_3_ solution in DMF (5 wt%) was spin-coated on PEDOT:PSS/ITO substrates with two steps (500 and 3000 rpm for 20 and 60 s, respectively). During the spin-coating process, 400-μL chlorobenzene (CB) was dropped onto these samples at countdown 40th second. Then, all of these samples were annealed at 100 °C for 10 min. TPBi about 40 nm was evaporated on top of perovskite film, followed by the deposition of Ag about 100 nm by thermal deposition in high vacuum condition. The overlap area between ITO anode and Ag cathode was 0.2 cm^2^, which was the active emission area of PeLEDs.

**Table 1:** Physical properties of polar solvents used in this study [30]

Chemical	Polarity (water = 100)	Dielectric constant (20 °C)	Solubility parameter (cal cm^3^)^1/2^	Boiling point/°C (water = 100)
MeOH	76.2	32.6	14.5	65
EtOH	65.4	22.4	13.4	78
IPA	54.6	18.3	11.5	82

### Device Characterization

The current density-voltage-luminance (*J-V-L*) characteristics were tested with a Keithley 4200 source. Electroluminescence (EL) spectra of PeLEDs were tested with a spectrophotometer OPT-2000. Device measurements were performed in air without encapsulation. Conductivity was measured by four-point probe technique with Hall Effect Measurement System (Suzhou Telecommunications Instrument Factory, SX 1934 (SZ-82)). Film thickness was measured by a step surface profiler. Surface morphologies of the PEDOT:PSS films and MAPbBr_3_ films were characterized by atomic force microscope (AFM; AFM 5500, Agilent, Tapping Mode, Chengdu, China). Crystallization of MAPbBr_3_ film was investigated by scanning electron microscopy (SEM; JEOL JSM-7100F). Crystal structure was characterized by X-ray diffraction (XRD; X’Pert PRO, PANalytical, Cu K*α*radiation *λ* = 0.154056 nm, 40 kV, and 40 mA). The time-resolved photoluminescence (TRPL) spectra were recorded by a time-correlated single-photon counting system (FL-TCSPC, Horiba Jobin Yvon) with 368 nm picosecond (10^−12^ s) pulsed laser. The statistics of the obtained luminescent parameters for PeLEDs provided in Additional file 1: Figure S1 which are consistent with the Gaussian Distribution, showing that the results are statistically significant and reproducible, providing a strong proof of the discussion.

## Results and Discussion

### Performance of PeLEDs

**Fig. 2:**
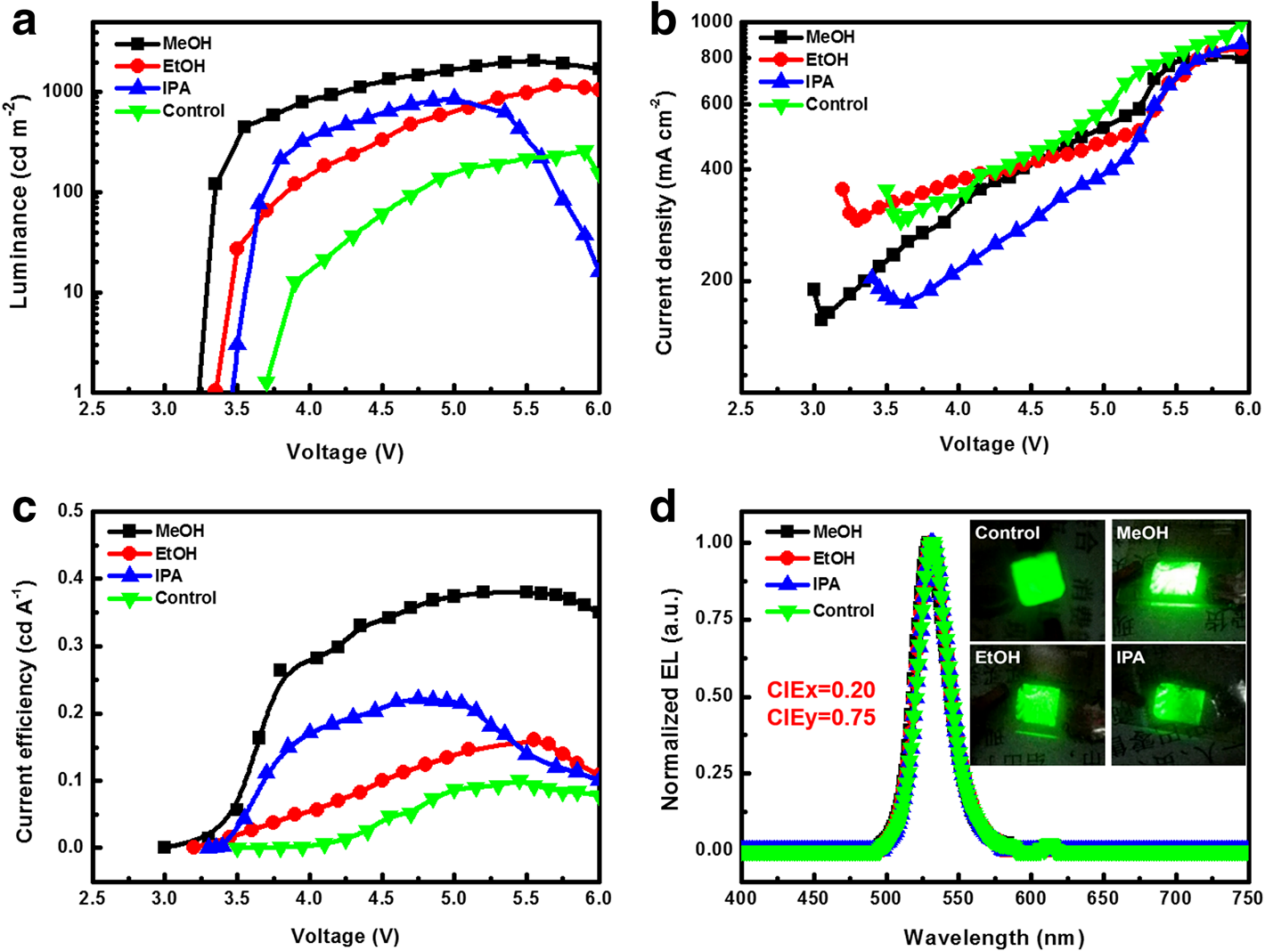
Device performance of PeLEDs. **a** Luminance-voltage (*L-V*) curves. **b** Current density-voltage (*J-V*) curves. **c** Current efficiency-voltage (*CE-V*) curves. **d** Normalized EL spectra and photographs of PeLEDs

Figure 2 shows the device performance with and without alcohols treating PEDOT:PSS films. And PeLED parameters, including maximum luminance (*L*_max_) and maximum CE (CE_max_) are summarized in Table 2. The control devices without alcohol solvent treatment show a *L*_max_ average of 261 cd m^−2^ and a CE_max_ average of 0.10 cd A^−1^. Compared to untreated devices, a higher *L*_max_ average of 2075 cd m^−2^ is achieved for MeOH-treated devices with a CE_max_ average of 0.38 cd A^−1^. The EtOH-treated devices have a *L*_max_ average of 1166 cd m^−2^ and CE_max_ average of 0.16 cd A^−1^, and the IPA-treated devices have a *L*_max_ average of 863 cd m^−2^ and CE_max_ average of 0.22 cd A^−1^. Obviously, the *L*_max_ of PeLEDs increase with the polarity of alcohol solvent increasing. We suspect that the improvement of device performance may be due to two reasons. One is that alcohol solvent treatment can facilitate the injection of hole into EML, and the other is that alcohol solvent treatment can promote the crystallization of MAPbBr_3_. As a result, the radiative recombination of excitons is enhanced. To verify the above postulation, the changes in PEDOT:PSS films and MAPbBr_3_ films are analyzed below.

**Table 2:** Performance of PeLEDs based on treated PEDOT:PSS films

HTL	Max. luminance (cd m^−2^ at bias)	Max. CE (cd A^−1^ at bias)
PEDOT:PSS	261 (at 5.90 V)	0.10 (at 5.90 V)
PEDOT:PSS-MeOH	2075 (at 5.55 V)	0.38 (at 5.55 V)
PEDOT:PSS-EtOH	1166 (at 5.70 V)	0.16 (at 5.70 V)
PEDOT:PSS-IPA	863 (at 5.10 V)	0.22 (at 5.10 V)

We also examined the EL characteristics of PeLEDs. As shown in Fig. 2d, at the voltage of 5.5 V, the EL emission peaks of all devices center at 532 nm with a FWHM about 27 nm. Meanwhile, the luminescent photographs of PeLEDs were tested at 6.0 V. There are no additional emission peaks in the EL spectrum, indicating that the emission of these PeLEDs comes from MAPbBr_3_ merely.

### Characterization of PEDOT:PSS Films

To illustrate the influence of alcohol solvent treatment on PEDOT:PSS films, the conductivity of PEDOT:PSS film is measured by a 4-point probe instrument. Conductivity values along with the pristine PEDOT:PSS films and after film treatment are shown in Table 3. As shown in Tables 1 and 3, the conductivity of PEDOT:PSS film increases with the enhancement of alcohol solvent polarity. Given this tendency, compared to 0.1 S cm^−1^ for pristine PEDOT:PSS film, the average conductivity values for PEDOT:PSS films treated with IPA and EtOH are 230.2 and 327.5 S cm^−1^, respectively. And for MeOH-treated films, an average conductivity of 605.0 S cm^−1^ can be achieved. It is well known that the Coulomb interaction between positively charged PEDOT and negatively charged PSS can be reduced by polar solvents [20]. Therefore, the alcohols with higher polarity is responsible for a stronger screening effect between PEDOT and PSS, so more amount of PSS are removed out with alcohols during the spin-coating process. As a result, the thickness of treated PEDOT:PSS film decreases, and the decline degree of film thickness varies with the polarity of alcohols solvent used. As shown in Table 3, the film thickness is 40 nm for untreated PEDOT:PSS layer, 27, 32, and 35 nm for MeOH-treated, EtOH-treated, and IPA-treated PEDOT:PSS films, respectively.

**Table 3:** Properties of the treated PEDOT:PSS films

HTL	Conductivity (S cm^−1^)	Thickness (nm)	RMS (nm)
PEDOT:PSS	0.1	40	2.53
PEDOT:PSS-MeOH	605.0	27	0.90
PEDOT:PSS-EtOH	327.5	32	1.85
PEDOT:PSS-IPA	230.2	35	1.97

**Fig. 3:**
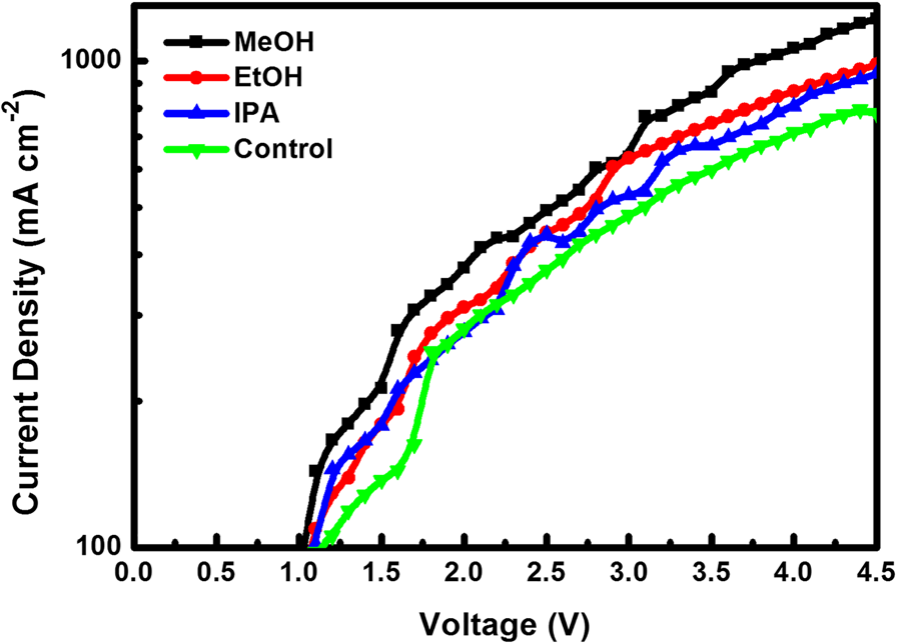
Current density versus voltage curves (CD-V) of hole-only PeLEDs with and without alcohol solvent treatments

To further characterize the hole injection capability of PEDOT:PSS films after alcohol solvent treatment, the hole-only devices with a structure of ITO/PEDOT:PSS/MAPbBr_3_ (70 nm)/MoO_3_ (30 nm)/Ag (100 nm) are fabricated and measured the hole current density, which is shown in Fig. 3. It is obvious that MeOH-treated device has the highest current density than the control device, EtOH- and IPA-treated devices, presenting that the higher of the solvents’ polarity, the greater of hole injection capability from PEDOT:PSS layer to EML.

**Fig. 4:**
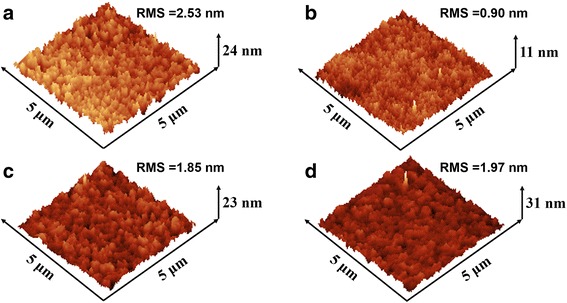
AFM morphology images of PEDOT:PSS films: **a** pristine PEDOT:PSS and **b**–**d** treated with MeOH, EtOH, and IPA, respectively

The AFM measurement is conducted to investigate the morphology changes of PEDOT:PSS film surface. Figure 4 shows the topography images of pristine and treated PEDOT:PSS films on ITO substrates. The root mean square (RMS) roughness of film decrease from 2.53 nm for pristine PEDOT:PSS film to 0.90, 1.85, and 1.97 nm for MeOH-treated, EtOH-treated, and IPA-treated PEDOT:PSS films, respectively. It can be seen that the morphology of treated PEDOT:PSS film is more uniform than the pristine PEDOT:PSS, and MeOH-treated film has the best optimized uniform than EtOH- and IPA-treated films.

### Characterization of MAPbBr_3_ Films

**Fig. 5:**
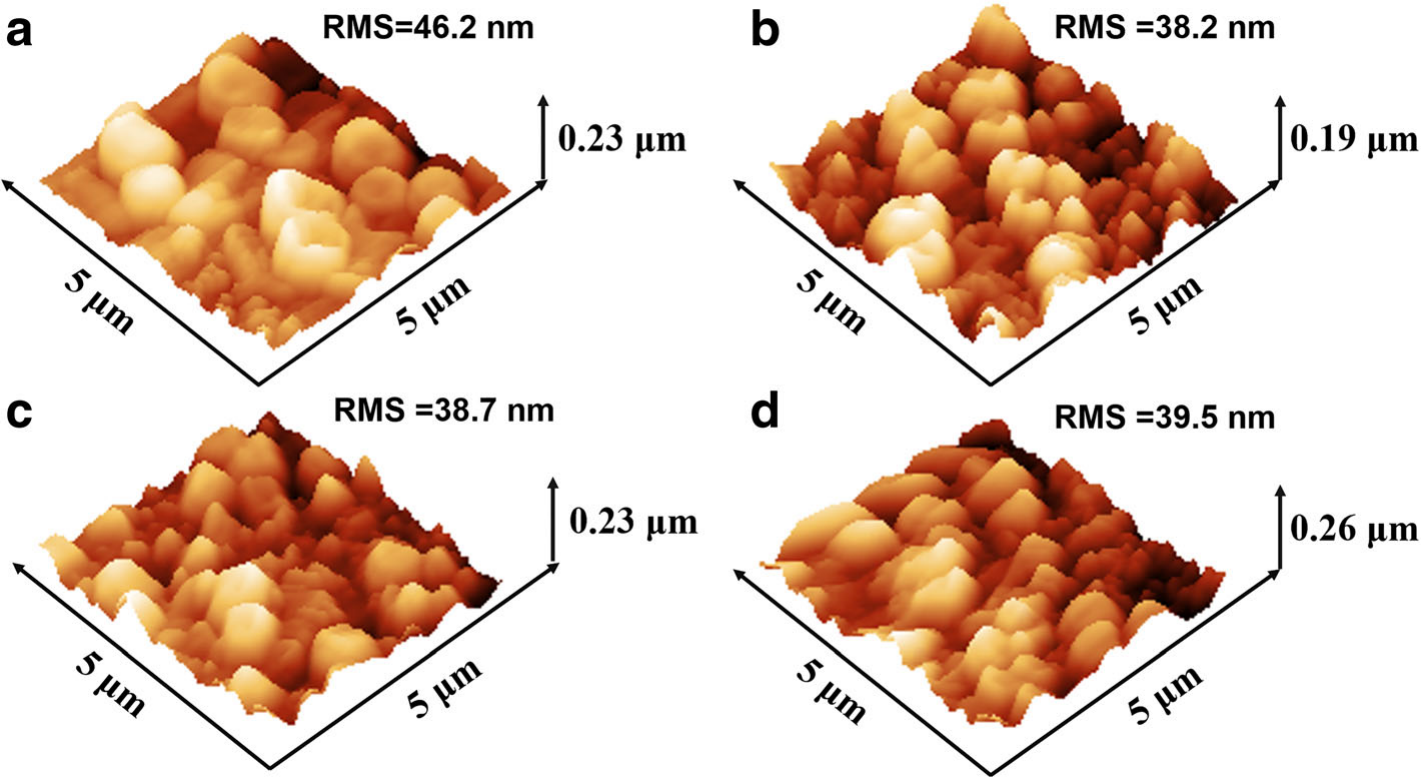
AFM morphology images of MAPbBr_3_ films: **a** based on pristine PEDOT:PSS film and **b**–**d** based on PEDOE:PSS films treated with MeOH, EtOH, and IPA, respectively

To investigate the effect of different alcohol treatment on MAPbBr_3_ film, the morphology and crystallization of MAPbBr_3_ are systematically studied. The AFM images of MAPbBr_3_ films based on PEDOT:PSS films treated with various alcohol solvent are shown in Fig. 5. For MAPbBr_3_ films based on pristine PEDOT:PSS films, the RMS roughness is 46.2 nm. And the RMS roughness of MAPbBr_3_ films decrease to 38.2, 38.7, and 39.5 nm for MeOH-treated, EtOH-treated, and IPA-treated PEDOT:PSS films, respectively. It can be seen that the decreased RMS roughness of MAPbBr_3_ films can smooth the MAPbBr_3_ films. And the RMS roughness of MAPbBr_3_ film decreases as the polarity of alcohol increases, which is consistent with the variation of PEDOT:PSS film’s RMS roughness.

**Fig. 6:**
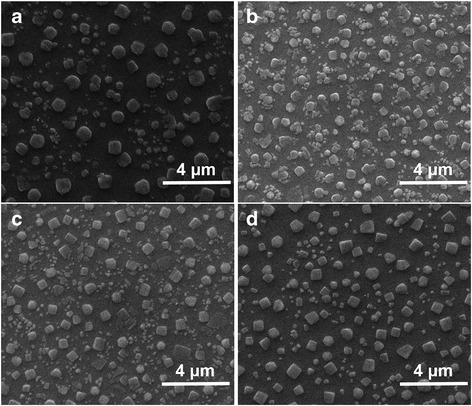
Top-view SEM images of MAPbBr_3_ films: **a** based on pristine PEDOT:PSS film and **b**–**d** based on PEDOT:PSS films treated with MeOH, EtOH, and IPA, respectively

To further confirm the grain size and coverage of MAPbBr_3_ films, a top-view scanning electron microscopy (SEM) is used, and the micrograph is shown in Fig. 6. Obviously, MAPbBr_3_ film based on MeOH-treated PEDOT:PSS film has the smallest grain size and best coverage. The average grain size is estimated by Image J (an imaging processing software) using SEM micrographs. The average grain size of MAPbBr_3_ decrease from 328.0 nm for MAPbBr_3_ based on pristine PEDOT:PSS films to 232.0, 252.9, and 272.8 nm based on MeOH-treated, EtOH-treated, and IPA-treated PEDOT:PSS, respectively. And the MAPbBr_3_ coverage increase from 24.95 to 37.34% for MeOH-treated, 33.0% for EtOH-treated, and 28% for IPA-treated, respectively. Additionally, there are many small grains around the large grains in MeOH group and EtOH group, but few in IPA group and control group. The reason for this phenomenon may be that the growth of larger MAPbBr_3_ grains at the expense of smaller grains is prevented. And the reason for this retarding effect is that the surface energy of PEDOT:PSS film increases, where MAPbBr_3_ grains grow on. The more uniform the PEDOT:PSS film, the bigger the curvature, which is responsible for a larger surface energy [25]. It can be demonstrated that the introduction of alcohol solvent with high polarity will increase the surface energy of the PEDOT:PSS film by forming more uniform film, thereby reducing the possibility of small grains ablation or large grains grow bigger. This phenomenon is very consistent with crystal growth as Ostwald ripening and can be easily observed in the case of quantum dots materials [25, 26]. From the above analysis, we can see that the method of alcohol solvent treating PEDOT:PSS films does enhance the crystallization of MAPbBr_3_.

**Fig. 7:**
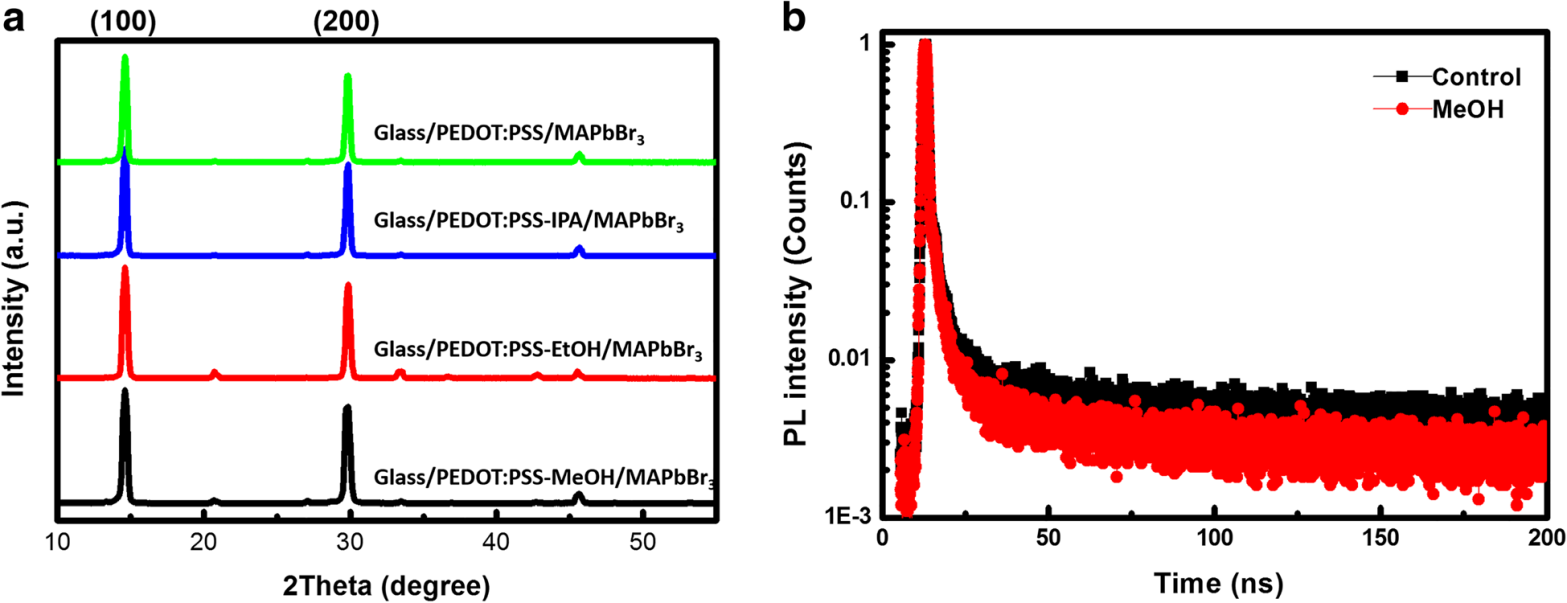
**a** XRD images of MAPbBr_3_ films and **b** time-resolved PL lifetime of MAPbBr_3_ films on PEDOT:PSS films with and without MeOH treatments

The crystal structure of MAPbBr_3_ film is analyzed by measuring X-ray diffraction (XRD) patterns, as shown in Fig. 7a. The films have two strong and sharp diffraction peaks at 14.602^o^ and 29.845^o^, corresponding to (100) and (200) planes, respectively. These two diffraction peaks are in good agreement with the previous report [27, 28], which demonstrates that MAPbBr_3_ crystals are highly oriented with a good cubic crystalline phase. To analyze the size of perovskite crystal, we can use Scherrer Equation as following:

1 $$ L=\frac{K\lambda}{B\cos \theta } $$

where *L* (nm) represents the crystallite size, *K* (0.89, spherical) represents the Scherrer constant, *λ* (0.154056 nm) represents the X-ray wavelength, *B* (rad) represents full width at half maximum of the XRD peak, and *θ* (rad) represents X-ray angle. Using Eq. (1), we calculate the perovskite crystallite size to be 32.5 ± 0.8 nm. With the change of alcohol solvent, the variation of crystallite size is negligible. This proves that the crystal structure of MAPbBr_3_ does not change after alcohol solvent treatment. As shown in Fig. 7b, TRPL decay curves of MAPbBr_3_ films based on PEDOT:PSS films with and without MeOH treatments have been recorded. The PL decay curves are well described by bi-exponential decay function, which contains a slow decay and a fast decay. The fast decay is related to trap-assisted recombination (i.e., non-radiative recombination), and the lower decay is related to radiative recombination [3, 29]. When using MeOH to treat PEDOT:PSS films, the PL lifetime of excitons decreases, indicating that in the condition of unchanged composition and crystal structure of MAPbBr_3_, the efficiency of radiative recombination increases. From the above discussion, we see that alcohol solvent treatment on PEDOT:PSS films could manipulate the grain size and the coverage of perovskite films, which has a clear correlation between the morphology of PEDOT:PSS film and crystallization of perovskite.

## Conclusions

In conclusion, alcohol solvent treatment on PEDOT:PSS films has been proposed to improve the luminance of PeLEDs. Compared to EtOH and IPA, MeOH solvent is the most appropriate to improve PeLEDs performance, resulting in a *L*_max_ of 2075 cd m^−2^ and a CE_max_ of 0.38 cd A^−1^. The luminance improvement can be attributed to the synergistic effect of alcohol solvent treatment. On the one hand, the higher the alcohol solvent polarity is, the more amount of PSS is taken away in the process of spin-coating alcohol solvent on PEDOT:PSS/ITO substrates. This will result in the higher conductivity of the treated PEDOT:PSS films, and more holes could be injected into perovskite active layer. On the other hand, the higher the alcohol polarity, the larger the surface energy of the PEDOT:PSS films, caused by their more uniform surface. The increased surface energy can restrain the Ostwald ripening and promote to grow smaller perovskite grains and better coverage, resulting in efficient radiative recombination. This provides that alcohol solvent treatment can be a valuable method to increase the baseline of PeLEDs performance, which will be widespread applicable in the future commercial production.

## Additional File

Additional file 1: **Figure S1.** Histogram of the maximum luminance of PeLEDs: (a) based on pristine PEDOT:PSS and (b–d) based on PEDOT:PSS films treated with MeOH, EtOH, and IPA, respectively. (DOCX 360 kb)

## Abbreviations

AFM: Atomic force microscope
CB: Chlorobenzene
CE: Current efficiency
CE_max_: Maximum current efficiency
EL: Electroluminescence
EML: Emission layer
EQE: External quantum efficiency
ETL: Electron transport layer
EtOH: Ethanol
FWHM: Full width at half maximum
HOMO: The highest occupied molecular orbital
HTL: Hole transport layer
IPA: Isopropanol
ITO: Indium tin oxide
*J-V-L*: The current density-voltage-luminance
*L*_max_: Maximum luminance
MAPbBr_3_: CH_3_NH_3_PbBr_3_
MeOH: Methanol
PEDOT:MoO_3_: Mixed PEDOT:PSS with MoO_3_
PEDOT:PSS: Poly(3,4-ethylenedioxythiophene):polystyrene sulfonate
PeLEDs: Perovskite light-emitting diodes
PFI: Perfluorinated ionomer
PLQY: Photoluminescence quantum yield
PMMA: Poly(methyl methacrylate)
RMS: Root mean square
SEM: Scanning electron microscopy
TPBi: 1,3,5-Tris(2-*N*-phenylbenzimidazolyl) benzene
TRPL: The time-resolved photoluminescence
XRD: X-ray diffraction
