# Supplementary material for: High-luminance perovskite light-emitting diodes with high-polarity alcohol solvent treating PEDOT:PSS as hole transport layer
Source: Nanoscale Res Lett. 2018 Apr 27;13:128. doi: 10.1186/s11671-018-2505-6 (PMC5919892; doi:10.1186/s11671-018-2505-6)
Supplement: Supplementary file 1 — Figure S1. Histogram of the maximum luminance of PeLEDs: (a) based on pristine PEDOT:PSS and (b–d) based on PEDOT:PSS films treated with MeOH, EtOH, and IPA, respectively. (DOCX 360 kb) [file 11671_2018_2505_MOESM1_ESM.docx]

**Supporting Information**

**High-luminance perovskite light-emitting diodes with alcohols solvent treating PEDOT:PSS as hole transport layer**

Mengge Wu, Dan Zhao, Zijun Wang, Junsheng Yu*

*State Key Laboratory of Electronic Thin Films and Integrated Devices, School of Optoelectronic Science and Engineering, University of Electronic Science and Technology of China (UESTC), Chengdu 610054, PR China*


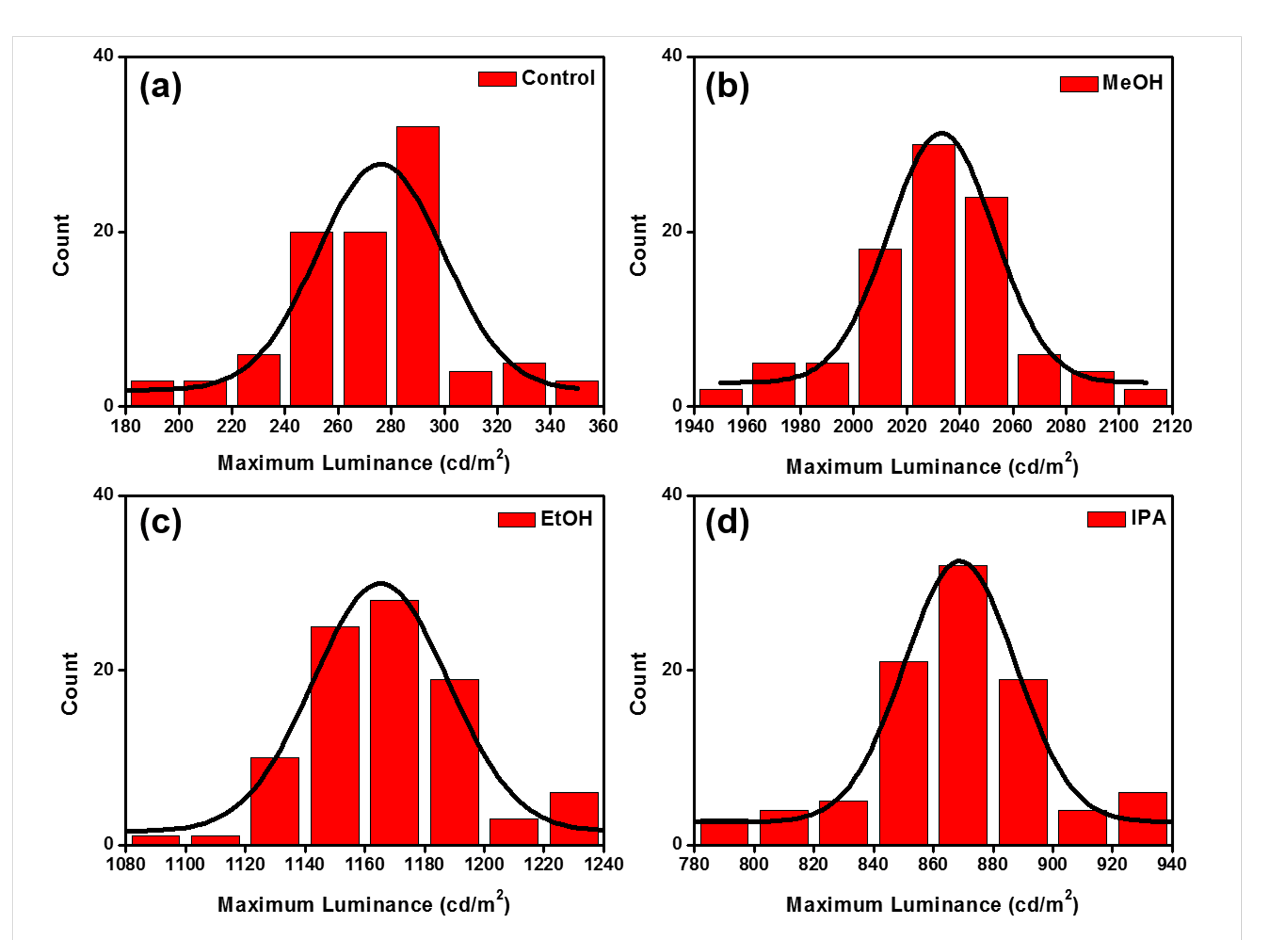


Fig. S1. Histogram of the maximum luminance of PeLEDs: (a) based on pristine PEDOT:PSS and (b-d) based on PEDOT:PSS films treated with MeOH, EtOH, IPA, respectively.
